# Undergraduate Skills Training in Pandemic Times: Where Is the Future of Medical Education?

**DOI:** 10.3390/ejihpe13070090

**Published:** 2023-07-07

**Authors:** Andrzej Hecker, Sebastian P. Nischwitz, Johanna Petritsch, Judith C. J. Holzer-Geissler, Alexander Draschl, Thomas Wegscheider, David Benjamin Lumenta

**Affiliations:** 1 Division of Plastic, Aesthetic and Reconstructive Surgery, Department of Surgery, Medical University of Graz, 8036 Graz, Austriadavid.lumenta@medunigraz.at (D.B.L.); 2 Research Unit for Digital Surgery, Division of Plastic, Aesthetic and Reconstructive Surgery, Department of Surgery, Medical University of Graz, 8036 Graz, Austria; 3 Clinical Skills Center (CSC), Medical University of Graz, 8036 Graz, Austria

**Keywords:** COVID-19, hybrid class, medical education, objective structured clinical examination (OSCE), online education, remote class, remote teaching, single interrupted suture, surgical training, surgical skills

## Abstract

Background: The COVID-19 pandemic forced medical programs to rapidly switch to remote teaching from scratch, impacting hands-on skills training. This study compared the efficacy of a hybrid online format to a regular in-person session for a mandatory surgical skills class. Methods: Third-year undergraduate medical students attending the surgical skills class in the winter semester of 2020/21 at the Medical University of Graz were randomly assigned to either the hybrid or in-person class, depending on their course schedule and government regulations. The hybrid class involved online videos, one-on-one peer tutoring, and an Objective Structured Clinical Examination (OSCE). Pre- and post-class self-assessments were conducted to evaluate their theoretical and practical knowledge of a single interrupted suture. Results: The study included 85 students in the regular in-person class and 50 in the hybrid class. A pre-class assessment revealed higher self-assessments in the hybrid class for theoretical and practical knowledge, but a post-class assessment showed no significant difference. The advantages and disadvantages of both modalities were identified, providing valuable insights for future curriculum development. Conclusions: Both teaching modes were effective for undergraduate surgical skills training. This study recommends implementing positive aspects of both the hybrid and in-person formats while recognizing their respective limitations.

## 1. Introduction

The 2020 pandemic due to COVID-19 precipitated significant disruptions to the social, educational, and occupational domains of numerous individuals [1,2,3,4]. In response to the COVID-19 pandemic, Austria implemented three statewide “strict lockdowns” between mid-March 2020 and March 2021. The initial lockdown was enforced on March 16 and persisted until April 2020. Subsequently, the second lockdown occurred from 17 November to 6 December 2020, followed by the third lockdown from 26 December 2020, to 7 February 2021 [5]. Throughout the pandemic, in response to the prevailing circumstances, various adjustments were implemented, aligned with moderate relaxations and a substantial decline in infection rates observed during the summer of 2020. Consequently, starting from the initial lockdown in March 2020, higher educational institutions, including medical schools, have consistently adopted distance learning [6,7]. Video conferencing and webinars had to be incorporated into daily workflows since lectures and in-person meetings had to be canceled or virtualized [8]. As a result, hybrid classrooms have been implemented at our medical school, allowing for a combination of online and in-person sessions whenever practical and allowed by Austrian legislative laws.

Technological advancements have brought about numerous changes in the field of medicine, including the nature of the interaction between educators and students [9]. With the outbreak of the COVID-19 pandemic, traditional methods of education that prioritize face-to-face interactions were quickly fully or partially replaced with hybrid classes or online learning [9]. This transition has required novel approaches and an infrastructure to facilitate effective education and training, with universities facing significant challenges in fulfilling their mandate to educate the next generation of medical professionals [10,11,12]. While online lectures and webinars have proven feasible for certain subjects, practical exercises that require in-person interaction, demonstration, and practice pose unique challenges [13].

The main objective of surgical education is to train skilled surgeons while encouraging personal development [14]. The surgical skills class, alongside the gross anatomy course, is among the most important introductory courses for surgeon recruitment [15,16,17]. Being proficient in performing a single interrupted stitch (SIS) is a fundamental component of surgical education and training, requiring theoretical and practical knowledge to be effectively learned [18]. Furthermore, it is crucial that not only surgeons, but all medical practitioners, can flawlessly perform this skill.

While online classes may not be sufficient to provide the necessary standardized learning experience for surgical skills [19], our faculty implemented a strategy to address the challenges imposed by federal regulations [20], introducing a combined online and in-person surgical skills course (hybrid). The course covered the theoretical basis of the SIS and advanced techniques via video lectures, followed by one-on-one practical sessions with trained student tutors.

The study aimed to compare the efficacy of the recently introduced hybrid model (hybrid) to the regular in-person suturing class.

## 2. Materials and Methods

This study aimed to evaluate the efficacy of a suture class held with social distancing measures in place, as compared to a conventional in-person class. As a result of the retrospective examination of prospective student evaluations of university courses, which are performed in the context of teaching, ethics committee approval was requested but waived. Participation in the study was voluntary, and the ethics committee raised no objections. The study was conducted in accordance with the Declaration of Helsinki and all other applicable and relevant regulations, which took place at the Medical University of Graz in Austria.

This study included all medical students who attended and evaluated the mandatory surgical skills class during their third year of medical school. At the time of this study, 659 medical students (53% women and 47% men) were enrolled in the third year of medical school (Medical University of Graz, Austria). In the winter semester of 2019/20, the number of medical students was 332 in total (53% women and 47% men).

The surgical suture class takes place multiple times per semester. In the winter semester of 2020/21, the allocation of students in either the hybrid online/small group (hybrid) or the conventional single-event group (in person) was not arbitrary. Instead, it was adjusted to comply with the federal government’s official regulations. While students were free to choose their preferred time slot, the method of course conduction was practically randomized based on the then-applicable government regulations. Following these regulations, universities, including the Medical University of Graz, transitioned to distance learning in mid-November 2020. Consequently, students were assigned to the hybrid course if their class took place after mid-November 2020, while those who had the course prior to that were allocated to the in-person course.

A questionnaire in German (Supplementary Figure S1), which was identical for both groups, was used to evaluate the anonymous and voluntary self-assessment of participants before and after the suturing class. The primary outcome measure for assessing the theoretical and practical knowledge of the SIS was conducted using a Likert Scale with five steps, ranging from 0 to 100. To assess these outcomes, the following questions (in German) were used for various suture techniques, including the SIS:

(1) Theoretical knowledge: How would you rate your theoretical knowledge of the following techniques (SIS)? (from 1 = very good to 5 = very poor)
(2) Practical knowledge: How would you rate your practical knowledge of the following techniques (SIS)? (from 1 = very good to 5 = very poor)

**Figure 1:**
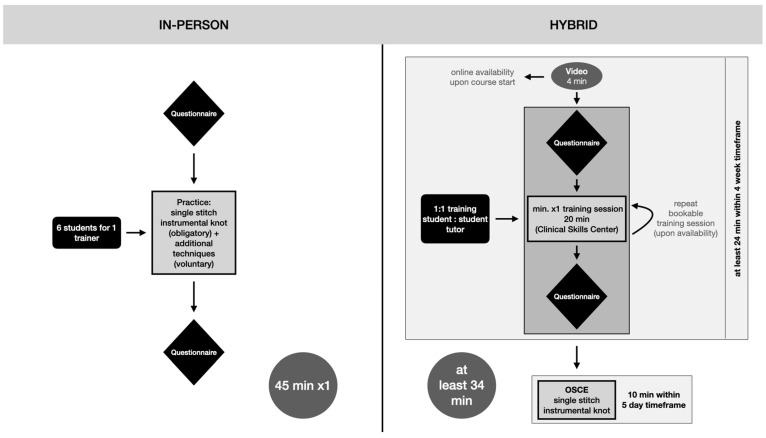
Modes of conduction for the surgical suture class during the winter semester of 2020/21.

The conduction mode differed depending on the student’s time slot during the winter semester of 2020/2021. The two methods of surgical skills classes are illustrated in Figure 1.

The regular in-person surgical skills class was conducted in German with a teacher/student ratio of 1:6 teacher (surgical resident/attending to student) and lasted 45 min. During the course, the fundamentals of surgical handling, the SIS technique, and advanced techniques were demonstrated in person and practiced together based on the students’ level of progress. The questionnaire (in German, Supplementary Figure S1) was handed out to the participants both at the beginning and at the end of the class.

The hybrid surgical skills class lasted at least 24 min, followed by a 10 min Objective Clinical Skills Evaluation (OSCE). After the questionnaire was handed out, the class began with an obligatory 4 min video that explained and demonstrated the basic surgical handling and technique of the SIS. During the mandatory 20 min one-on-one practice session, students were paired with a trained student tutor from a higher semester in a 1:1 ratio. Students were allowed to repeat the practice session voluntarily, subject to tutor availability. The hybrid class concluded with a 10 min OSCE assessment within five days of the course.

Data were analyzed using GraphPad Prism software (version 9.0.2; GraphPad Software, Inc., San Diego, CA, USA). Descriptive statistics are indicated using medians and 25th (Q1) and 75th (Q3) quartiles, while the Wilcoxon rank sum test and Mann–Whitney test were used for inferential statistics. For power analyses (G*Power 3.1.9.7.) of self-reported student confidence, an effect size of 0.5, an alpha level of 5%, and a power of 0.80 was used and the minimum sample size was 52 students per group [21]. The effect size was expressed as Cohen’s d and was categorized as small (d = 0.2), medium (d = 0.5), or large (d ≥ 0.8) [22]. All statistical tests were two tailed. The level of significance was set to *p* < 0.05. To facilitate data interpretation, means (MN) and standard deviations (SD) are additionally indicated the first-time data are presented.

## 3. Results

A total of 135 students participated in this study, with 50 students attending the hybrid class and 85 attending the in-person course. Table 1 presents the advantages and disadvantages of both classes, which are derived from a summary of free-text survey comments obtained from our questionnaire (Supplementary Figure S1), along with the authors’ opinions on the investigated hybrid and in-person course modalities.

### 3.1. Within Comparison

**Figure 2:**
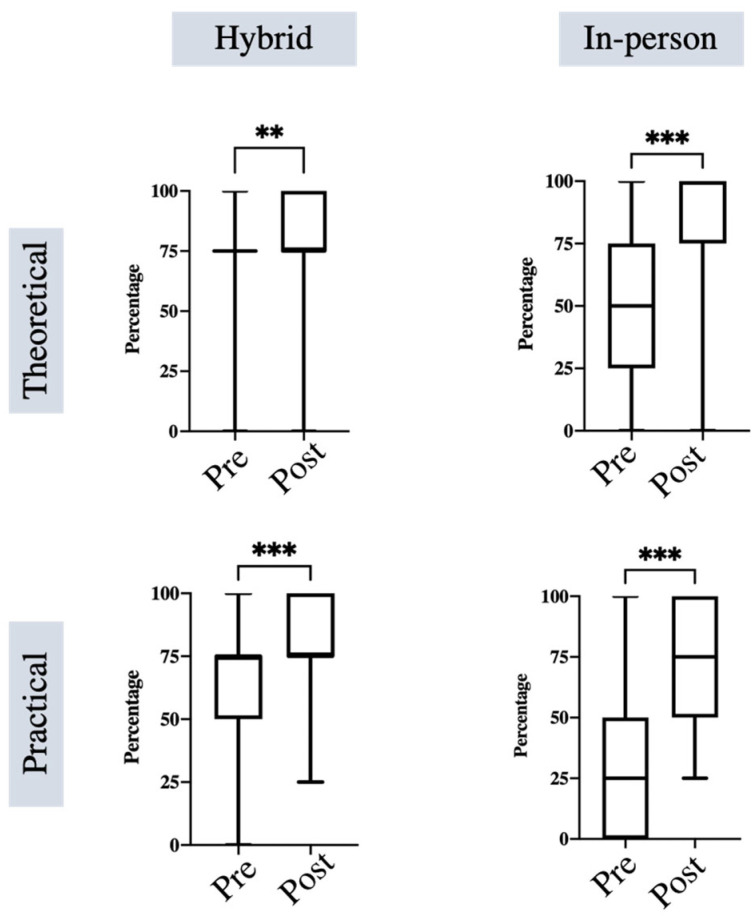
The figure displays the results of the within-group comparison, evaluating the effectiveness of both the hybrid and in-person course modalities for theoretical and practical knowledge of the SIS (single interrupted suture) before and after the course. The boxplots represent the medians (middle line), the 25th and 75th quartiles (box), and the total range. ** *p* = 0.002; *** *p* < 0.001.

The comparative analysis of theoretical and practical knowledge of the SIS within the hybrid and in-person classes before and after the course is presented in Figure 2.

#### 3.1.1. Hybrid Class

The students enrolled in the hybrid class provided a median rating of 75 (Q1: 75, Q3: 75; MN: 72.50, SD: 22.16) for their theoretical knowledge of the SIS before the course and a rating of 75 (Q1: 50, Q3: 75; MN: 61.00, SD: 27.27) for their practical knowledge. Following the class, their theoretical knowledge was rated at 75 (Q1: 75, Q3: 100; MN: 82.00, SD: 19.59), and their practical knowledge at 75 (Q1: 75, Q3: 100; MN: 78.50, SD: 17.50). The difference between the ratings of the theoretical and practical assessments before and after the course within the groups was statistically significant (*p* = 0.002, d = 0.45 and < 0.001, d = 0.79, respectively).

#### 3.1.2. In-Person Class

In-person class students rated their initial theoretical knowledge of the SIS at a median of 50 (Q1: 25, Q3: 75; MN: 42.94, SD: 32.42) and their practical knowledge at 25 (Q1: 0, Q3: 50; MN: 37.35, SD: 31.25). After the class, the theoretical knowledge was rated at 100 (Q1: 75, Q3: 100; MN: 82.06, SD: 23.97), and the practical knowledge at 75 (Q1: 50, Q3: 100; MN: 76.76, SD: 22.09). The difference in theoretical and practical assessments was significant (*p* < 0.001, d = 1.37 and *p* < 0.001, d = 1.46, respectively).

### 3.2. Comparison between Hybrid Class and In-Person Class

This section presents the comparative analysis of theoretical and practical knowledge of the SIS between the hybrid and in-person classes before and after the course.

#### 3.2.1. Pre-Class

The students enrolled in the hybrid class assessed their theoretical knowledge of the SIS at 75 (Q1: 75, Q3: 75), while those in the in-person class rated their knowledge at 50 (Q1: 25, Q3: 75). In terms of practical knowledge, the hybrid class students rated themselves at 75 (Q1: 50, Q3: 75). In contrast, the in-person class students rated themselves at 25 (Q1: 0, Q3: 50). The differences in both pre-class assessments were statistically significant (*p* < 0.001 for both). 

#### 3.2.2. Post-Class

The hybrid class students rated their theoretical knowledge at a median of 75 (Q1: 75, Q3: 100), while in-person class students rated theirs at a median of 100 (Q1: 75, Q3: 100). The practical knowledge was rated at 75 (Q1: 75, Q3: 100) in the hybrid class and 75 (Q1: 50, Q3: 100) in the in-person class. However, the post-class assessments’ theoretical and practical knowledge differences were not statistically significant (*p* = 0.51 for theoretical and *p* = 0.82 for practical knowledge). 

## 4. Discussion

The objective of this investigation was to compare the efficacy of a regular in-person suture class to a hybrid suture class consisting of observational practice (video and a one-on-one session), feedback learning (one-on-one session), and self-controlled practice (video) via self-assessment tests using the Likert Scale before and after completion of the course. 

We could show a significant increase in both theoretical and practical knowledge of the SIS after both classes, assuming effective learning in both groups. Notably, the pre-class evaluation was significantly higher for theoretical and practical knowledge in the hybrid group than in the in-person group, implying that hybrid class participants may have had a higher self-motivation to learn the topic in a more self-dependent mode of conduction as compared to the traditional teacher-centered teaching [23]. This may be due to the opportunity for hybrid class students to prepare the theoretical background through online videos before the class, which may have led to higher self-assessment ratings in theoretical and practical knowledge. The shift from teacher-centered to learner-centered learning may explain the better pre-class evaluations with an assumed stronger incentive to prepare for the class [24,25].

The results of the post-class evaluation revealed no statistically significant differences in theoretical and practical knowledge of the SIS between hybrid and in-person classes. Therefore, our study suggests that implementing the hybrid model for surgical skills training can be just as effective as traditional in-person classes while adhering to educationally challenging regulations without requiring additional budget-draining resources. With a Cohen’s d of 1.37 (theoretical knowledge) or 1.46 (practical knowledge) in the in-person course, there was a large practical significance. In contrast, the hybrid course presented a small to medium practical significance (theoretical knowledge: d = 0.49; practical knowledge: d = 0.79). These results suggest that the in-person course was more effective compared to the hybrid course. It is worth noting that students of the hybrid class entered the study with more confidence than students of the in-person class. This may have affected our results. Moreover, the educational experience may also be impacted by how students are assigned to courses in accordance with governmental regulations. Considering that both cohorts are comparable to a limited extent, our findings should be interpreted with relevant caution. 

Our findings are consistent with those of Co et al. [26], who found that medical students who attended web-based surgical skills learning (case group) and face-to-face learning (control group) had comparable clinical competency assessments. Their study participants were final year medical students on surgical rotation. Our hybrid group differed from Co et al.’s case group in that they were provided with a video lecture in addition to an in-person session, without mandatory hands-on practice by the students or the distribution of equipment for self-controlled training of the surgical skill before the face-to-face session. In addition, our study population consisted of third year medical students. Despite these differences, previous research [27,28,29,30,31] has demonstrated that various types of online learning, including the implementation of augmented reality (AR) [32,33], can be a feasible and acceptable approach to learning medical content and surgical skills in general. However, further studies are necessary to determine whether there is an optimal mode of delivery for surgical education that outperforms traditional methods.

A relevant aspect promoting the hybrid theory is the fact that the video learning module for acquiring the theoretical knowledge was always available during the period of the course, which allowed for a flexible schedule and commitment to the class and provided an opportunity for students to review unclear surgical steps when necessary. Furthermore, since clinical personnel are often engaged in various daily tasks, including scientific and clinical responsibilities, creating the video content just once is a time-efficient approach for the teachers. Bochenska et al. [34] demonstrated that a teaching video had a positive effect on medical student’s ability to tie surgical knots. The obligatory 20 min one-on-one peer-controlled session further facilitated student engagement and allowed for specific problem solving. The respective feedback of the tutor was directly focused on the learner’s needs in a bi-directional manner, and the fact that the tutor was one of the learner’s peers most likely created a positive learner–teacher relationship, thus maximizing the learning benefit [35,36,37,38]. Finally, our hybrid model combined observational practice (a video and a one-on-one session), feedback learning (one-on-one session), and self-controlled practice (video), which are known to be highly influential factors in motor skill learning [39]. Additionally, the OSCE provided a stressor for students and probably motivated them to demonstrate their acquired skills under controlled circumstances, which likely led to even more effective learning [40].

The forced adaptation to the hybrid model provided an opportunity to evaluate its advantages and disadvantages rapidly. However, one major drawback of the hybrid class was its limited flexibility in terms of content, preventing students who were particularly interested in the topic from further developing their skills beyond the basic class. Furthermore, students whose interest was awakened by the class could not intensify their skills within the scope of this basic class, as the tutors with whom the students could practice were only trained in a standardized way for the SIS. Additionally, students expressed dissatisfaction with the limited individual one-on-one time during the traditional in-person bulk sessions, most likely owing to the 1:6 teacher/student ratio. However, the feasibility of increasing the one-on-one teaching time was constrained by the high ratio of students per surgeon, which exceeded the capacity of a concise 45 min class session. Nevertheless, during these sessions, opportunities for applying for electives in surgical divisions were openly promoted. 

Following this investigation, we have discerned the benefits and drawbacks of each modality. Our results suggest that the hybrid model is an effective and successful educational approach for teaching basic surgical skills such as the SIS. Due to the lack of objective performance data, the results of our study can only be considered for pedagogical effectiveness and not actual clinical performance. Therefore, future studies should additionally address performance data (e.g., OSCE results). Hereby, associations between course modalities, students’ performance regarding suture techniques (e.g., SIS), and subjective confidence can be evaluated. While the hybrid instruction model is still accessible to medical students via a web-based platform associated with Medical University, our school returned the hybrid class to its pre-pandemic modality. As a result, our medical students have the option to supplement their pre-pandemic modalities with hybrid education in a non-mandatory fashion. However, it is essential to provide a more personalized learning experience with experienced surgeons in order to advance to more intricate suturing and knotting techniques. Nonetheless, our findings contribute to the growing body of literature highlighting the importance of implementing standardized and readily available media to enhance medical education.

## 5. Limitations

One of this study’s limitations is that the evaluation of the teaching modalities was based on self-assessment without an objective performance assessment, which may be subject to biases. Thus, no association with actual clinical performance (SIS) can be made. Furthermore, heterogenous cohorts regarding pre-interventional confidence and timing of the intervention (before/after government regulations) lead to a limited comparability. 

Another limitation is the narrow time frame of evaluation, which did not account for the potential effects on (a) the motivation to practice at home and (b) long-term knowledge retention. Additionally, individual factors such as concentration, mood, emotions, and other aspects that could have influenced the students’ self-assessment were not assessed. Furthermore, the study design did not allow for the differentiation between the effects of the video, the one-on-one session, the OSCE, or other confounding factors. However, these adaptations were necessary to maintain a comprehensive medical education while adhering to government regulations. Therefore, we emphasize that the results of this study should be considered complementary rather than conclusive.

## 6. Conclusions

The unforeseeable recurrence of the COVID-19 pandemic, along with government regulations, have compelled universities to shift a significant portion of their education to remote learning. When the efficacy of two distinct teaching modes for teaching surgical skills as part of undergraduate medical education was evaluated, the in-person course outperformed the hybrid course in terms of practical significance. Through cohort-based limitations, the hybrid online/one-on-one suture course, implemented in response to the pandemic, is comparable to a limited extent to the bulk in-person class conducted prior to any regulations. Moreover, our findings are based on students’ self-assessments without objective performance evaluations. Therefore, rather than being deemed conclusive, our data results should be evaluated with appropriate caution. However, given the need for educational programs to adapt to pandemic conditions, we highly encourage future programs to implement standardized media, such as videos, into curricula whenever possible and appropriate. Furthermore, future large-scale studies should examine the impact of individual factors on learning efficacy.

## Figures and Tables

**Table 1:** Advantages and disadvantages of hybrid/online and in-person suture for medical students.

	Pro	Contra
**Hybrid ^1^**	Flexible schedule	Limited flexibility (pre-made videos, peers trained only for a certain set of skills)
	Unlimited video availability	
	Repeatable 20 min one-on-one session	No contact with experienced surgeons
	Peer practice session with direct feedback	No advanced surgical skills training
	Peer-to-peer teaching	
	OSCE as motivation	
	Pandemic conforming	
**In-person**	Individual help from experienced surgeons	Resource binding (time and personnel)
	Contact to surgeons allowing learning from “pros” and “mingling” in a risk-free teaching environment	Limited organizational flexibility
	Valuable recruitment base of surgical divisions for interested medical students	Limited time to teach everyone to everyone

^1^ Hybrid class consisting of observational practice (video and a one-on-one session), feedback learning (one-on-one session), and self-controlled practice (video).

## Data Availability

The datasets used and analyzed during the current study can be obtained from the corresponding author upon reasonable request.

## Supplementary Materials

The following supporting information can be downloaded at: https://www.mdpi.com/article/10.3390/ejihpe13070090/s1, Figure S1: Questionnaire.
