# Supplementary material for: Undergraduate Skills Training in Pandemic Times: Where Is the Future of Medical Education?
Source: Eur J Investig Health Psychol Educ. 2023 Jul 7;13(7):1219–28. doi: 10.3390/ejihpe13070090 (PMC10377890; doi:10.3390/ejihpe13070090)
Supplement: Supplementary file 1 [file ejihpe-13-00090-s001.zip › ejihpe-2424250-supplementary.pdf]

Fragebogen – pre Kurs

1. **Ich bin im … Studienjahr.**

☐ 1. ☐ 2. ☐ 3. ☐ 4. ☐ 5. oder höheren

2. **Nach Abschluss meines Medizinstudiums strebe ich eine Laufbahn…**

☐ … in einem operativen Fachgebiet an.
☐ … in einem nicht-operativen, klinischen Fachgebiet an.
☐ … in einem nicht-klinischen Fachgebiet an.
☐ … in keiner der genannten Richtungen an.

3. **Bis zum heutigen Tag habe ich … Wochen in operativen Fachgebieten famuliert.**

☐ 0 ☐ 1-4 ☐ 5-8 ☐ 9-12 ☐ 12-16 ☐ >16

4. **Wie schätzen Sie Ihre theoretischen Kenntnisse der folgenden Techniken ein? (1 = Sehr gut – 5 = Sehr schlecht)**

Einzelknopfnaht	☐ 1	☐ 2	☐ 3	☐ 4	☐ 5
Fortlaufnaht	☐ 1	☐ 2	☐ 3	☐ 4	☐ 5
Knüpfen	☐ 1	☐ 2	☐ 3	☐ 4	☐ 5
Gefäßligatur	☐ 1	☐ 2	☐ 3	☐ 4	☐ 5

5. **Wie schätzen Sie Ihre praktischen Fertigkeiten der folgenden Techniken ein? (1 = Sehr gut – 5 = Sehr schlecht)**

Einzelknopfnaht	☐ 1	☐ 2	☐ 3	☐ 4	☐ 5
Fortlaufnaht	☐ 1	☐ 2	☐ 3	☐ 4	☐ 5
Knüpfen	☐ 1	☐ 2	☐ 3	☐ 4	☐ 5
Gefäßligatur	☐ 1	☐ 2	☐ 3	☐ 4	☐ 5

Fragebogen – post Kurs

1. **Wie schätzen Sie Ihre theoretischen Kenntnisse der folgenden Techniken ein? (1 = Sehr gut – 5 = Sehr schlecht)**

Einzelknopfnaht	☐ 1	☐ 2	☐ 3	☐ 4	☐ 5
Fortlaufnaht	☐ 1	☐ 2	☐ 3	☐ 4	☐ 5
Knüpfen	☐ 1	☐ 2	☐ 3	☐ 4	☐ 5
Gefäßligatur	☐ 1	☐ 2	☐ 3	☐ 4	☐ 5

2. **Wie schätzen Sie Ihre praktischen Fertigkeiten der folgenden Techniken ein? (1 = Sehr gut – 5 = Sehr schlecht)**

Einzelknopfnaht	☐ 1	☐ 2	☐ 3	☐ 4	☐ 5
Fortlaufnaht	☐ 1	☐ 2	☐ 3	☐ 4	☐ 5
Knüpfen	☐ 1	☐ 2	☐ 3	☐ 4	☐ 5
Gefäßligatur	☐ 1	☐ 2	☐ 3	☐ 4	☐ 5

3. **In welchem Rahmen würden Sie die erworbenen Techniken am ehesten vertiefen?**

☐ Weitere Pflichtlehre
☐ Wahlfächer
☐ Praktika (z.B. Famulaturen)
☐ Freizeit

4. **Denken Sie an den theoretischen Nahtkurs zurück. Hat sich Ihr Verständnis über die gezeigten Techniken gefestigt? (1 = Auf jeden Fall – 5 = kein bisschen)**

☐ 1 ☐ 2 ☐ 3 ☐ 4 ☐ 5

5. **Was hat Ihnen besonders gut/schlecht gefallen? Was fehlt Ihnen? (Optional) [Freitext]**
